# Managed Entry Agreements for Oncology Drugs: Lessons from the European Experience to Inform the Future

**DOI:** 10.3389/fphar.2017.00171

**Published:** 2017-04-04

**Authors:** Kim Pauwels, Isabelle Huys, Sabine Vogler, Minne Casteels, Steven Simoens

**Affiliations:** ^1^Department Pharmaceutical and Pharmacological Sciences, KU LeuvenLeuven, Belgium; ^2^World Health Organization Collaborating Centre for Pharmaceutical Pricing and Reimbursement Policies, Gesundheit Österreich GmbHVienna, Austria

**Keywords:** cancer, managed entry agreements, risk sharing, reimbursement

## Abstract

**Objectives:** The aim of this study is to conduct an analysis on the regulation and application of managed entry agreements (MEA) for oncology drugs across different European countries.

**Methods:** Literature search and document analysis were performed between September 2015 and June 2016 to collect information on the regulatory framework and practice of MEA in Belgium, The Netherlands, Scotland, England and Wales, Sweden, Italy, Czech Republic and France. An overview of the content and typology of MEA applied for oncology drugs between 2008 and 2015 was generated based on publically available sources and contributions by national health authorities. Semi-structured interviews were conducted with representatives of national health authorities involved in the management or negotiation of MEA.

**Results:** The application of MEA differs across countries and across different indications for the same drug. Financial based agreements are prevailing due to their simplicity compared to performance-based agreements. Performance-based agreements are less commonly applied in the European countries except for Italy. In the Netherlands, application of performance-based agreements was stopped due to their inability to deal with dynamics in the market, which is highly relevant for oncology drugs.

**Conclusions:** MEA constitute a common policy tool that public payers in European countries use to ensure early access to highly priced oncology drugs. In light of strengths and weaknesses observed for MEA and the expected developments in the oncology area, the importance of MEA is likely to grow in the future.

## Introduction

Marketing authorization, price setting and reimbursement of drugs have to maximize accessibility and affordability of safe and effective drugs. The oncology domain faces important challenges for market access since the balance between harm and benefits is difficult for aggressive treatments such as chemotherapy and radiotherapy. While recent targeted therapies more selectively strike disease targets, benefits are often hard to measure in a clinically relevant way within the limited timeframes of clinical studies. Over the last decade, health systems were increasingly burdened with fast growing expenditures on oncology drugs, with a total expenditure in Europe from 7.6 billion in 2005 to 19.1 billion in 2014 (Jönsson et al., 2016). Uncertain benefit-risk ratios put the high prices for oncology drugs under debate (Garattini and Bertele, 2002; Fojo and Grady, 2009; Abboud et al., 2013; Pauwels et al., 2014; Fojo and Lo, 2016; Gozzo et al., 2016).

The European Commission (EC) can grant marketing authorization (MA) after the European Medicines Agency (EMA) approves the quality, safety and efficacy of the drug, requiring applicants to provide solid grounds on these requirements^1^. When the benefit of immediate availability outweighs the risk of incomplete data, conditional marketing authorization can however be granted in the interest of public health. A pilot project on adaptive pathways explored the implications of progressive patient access in parallel with the development of the drug, applied within the current regulatory framework for marketing authorization in areas of high medical need. Through adaptive pathways, conditional approval can be granted to a restricted patient group based on early data, while its use will enable real-life evidence generation that can be considered to broaden the approved patient population. Of the 62 proposals for the adaptive pathways pilot project, anti-neoplastic agents were the leading group of therapeutic agents (33%) (Eichler et al., 2015).

Trends toward early marketing authorization have potential benefits for patients, but also limit data availability for pricing and reimbursement decisions. Each European Member State takes into account its own regulations and economics for price setting and reimbursement, leading to heterogeneity in pricing, reimbursement and access to oncology drugs across Europe (Pauwels et al., 2014; Vogler et al., 2015)^2^. Managed entry agreements (MEA) are contractual agreements between the marketing authorization holder (MAH) and health care payers that are introduced when decisive “yes” or “no” conclusions on price and reimbursement could not be made due to uncertainties about the clinical evidence and/or financial impact of the drug (Carlson et al., 2009). Several countries apply MEA and different terminology and typology is identified, but the common factor is that MEA allow market access of drugs by sharing the cost of uncertainty between the payer and the MAH. MEA are an attractive mechanism for market access of oncology drugs (Ferrario and Kanavos, 2015; van de Vooren et al., 2015; Executive Insight Health Care Consultants, 2016). The aim of this study is to conduct a comparative analysis of the regulation and application of MEA for oncology drugs between European countries. Given current evolutions that will further complicate market access decision for oncology drugs, the experiences from the past will be investigated to draw lessons for the future application of MEA for oncology drugs.

## Methods

Literature search and document analysis was performed between September 2015 and June 2016 to collect information on the application of MEA in Belgium, the Netherlands, Scotland, England and Wales, Sweden, Italy, Czech Republic and France. Selection of countries was based on geographical distribution and variety in financing of health care system (compulsory health insurance vs. general taxation) and organization of health care system (National Health Service vs. insurance based). Literature was searched in PubMed and EMBASE based on but not limited to the following search terms: managed entry agreement(s), risk-sharing agreement(s), patient access scheme(s), performance-based reimbursement, coverage with evidence, price-volume agreement, discount, conditional reimbursement. The websites of national health authorities and organization involved in research related to health policy such as International Society for Pharmaco-economics and Outcomes Research (ISPOR) were consulted. Information was further refined through semi-structured interviews with the relevant authorities involved in the set-up and negotiation on MEA, including selected members of the Pharmaceutical Pricing and Reimbursement Information (PPRI) network. The interviews were conducted by telephone and are audio-recorded, ad verbatim transcribed and analyzed using the grounded theory approach. A country-specific summary on the results was presented to the national health authorities for validation. For each country, an overview of MEA applied at national level between 2008 and 2015 (including MEA set up since 2008 but expired during this period), for drugs intended to treat solid tumors or hematological malignancies, was generated. Agreements focusing on measures for appropriate use only, were not included. A reporting template was designed to include the drug name, substance name of the MAH, ATC code, indication, start-date of MEA, dates of prolongation of MEA and predicted end-date of MEA. All MEA were assigned to the typology described by Morel et al. (2013). If information could not be retrieved from publically available sources or was incomplete, it was requested from the national competent authorities. In case that information could not be disclosed due to confidentiality, descriptive numbers on the typology of MEA applied in the country of interest were requested. Descriptive analysis on the dataset was performed in Excel.

## Results

A country specific overview about the institutional and legal framework valid for MEA, as well as a comprehensive overview of the content of MEA for which data were retrieved, can be found in Supplementary Information.

For Belgium, Scotland, Sweden, England, Wales, and Italy, information about 164 specific MEA was collected. Information on MEA applied in Belgium, Sweden and Italy was obtained from health authorities, while publically available sources were available for England, Wales and Scotland. The 164 MEA identified in this study, involve 58 individual drugs. Majority of identified MEA was applied by Italy (63/164), followed by Scotland (43/164), England (28/164), and Belgium (28/164).

For the Czech Republic, 19 MEA are applied for oncology drugs. Descriptive information was obtained from the Insurance Funds Union. In France, the Ministry of Social Affairs and Health indicated that all information about MEA is strictly confidential. Literature indicated the existence of two MEA for oncology drugs^3^. In the Netherlands, the application of MEA was under transition at the time of this study and by 2016, an agreement was concluded for an oncology drug (nivolumab) for the first time since the reform.

### Characteristics of the drugs

Considering all MEA for which information about drug characteristics was retrieved (*N* = 164), a third of MEA (47/164) applies to Orphan Medicinal Products (OMP). Hematology is the leading disease area subjected to MEA (74/164). Detailed information on the indications of the drugs that are subjected to these MEA can be found in Supplementary Information.

Forty out of 164 MEA involve a drug that is subjected to a MEA in two or more of the countries considered, but the content of the schemes varied across countries.

Bevacizumab was the drug with the highest number of MEA, i.e., 10 MEA were applied across three different countries and six different indications. Italy applied a MEA for five different indications of bevacizumab and the type of scheme differs between different indications. This is illustrated by a financial based agreement for first line treatment of colorectal cancer, while a performance-based agreement is applied for second line treatment of this disease. Also in Belgium, the agreement can be differentiated across indications.

For only two drugs, enzalutamide (Xtandi®) and aberitaterone acetate (Zytiga®), MEA were applied in all six countries considered for data collection. Both drugs are used in prostate cancer and also in this case, Belgium and Italy distinct the MEA based on first and second line indication. For Xtandi®, all countries applied financial based agreements. For Zytiga®, a performance-based agreement was applied in Italy, while other countries applied financial-based agreements.

For Czech Republic and France, no complete information on characteristics of diseases covered by MEA could be obtained.

### Characteristics of the schemes

For Italy, England, Wales, Scotland and Sweden, specific information on the content of schemes was retrieved (*N* = 136) (Supplementary Information). Financial agreements were prevailing, with only one third of schemes (40/136) having a performance-based component. Except for three performance-based agreements applied in England, Wales and Scotland, all these MEA with a performance-based components were applied in Italy. As performance-based agreements are rare or inexistent in the majority of countries included in this study, we cannot link the typology of the schemes to the typology of the drugs that are involved.

In the Netherlands, performance-based agreements were applied in the past but the system is now under reform to only apply financial agreements. In France, a performance-basedcontract was signed for Imnovid® in 2014, indicated to treat multiple myeloma^3^.

### Transparency of the agreements

For Belgium and France, the content of MEA is strictly confidential for all parties who are not involved in the negotiation.

In the Czech Republic, the contents of MEA are confidentially negotiated between MAH and the Insurance Funds Union, who is responsible for reimbursement. The details of the scheme are not disclosed to the State Institute of Drug Control, who is responsible for price setting of drugs.

Information on medicines subject to a MEA is public information in Italy, England, Wales and Scotland and Sweden. Confidentiality of financial details is however the cornerstone of the application of MEA irrespective of the country where the MEA is applied. The Pharmaceutical Price Regulation Scheme (PPRS) that is valid in United Kingdom (UK) states that all MEA should be transparent, with an exception when the Ministers have agreed that the details of the discount can remain commercial-in-confidence. When a medicine subject to an agreement is used as a comparator for the appraisal of another treatment, relevant details of the MEA need to be available to relevant consultees and commentators.

### Financial-based agreements

**Figure 1:**
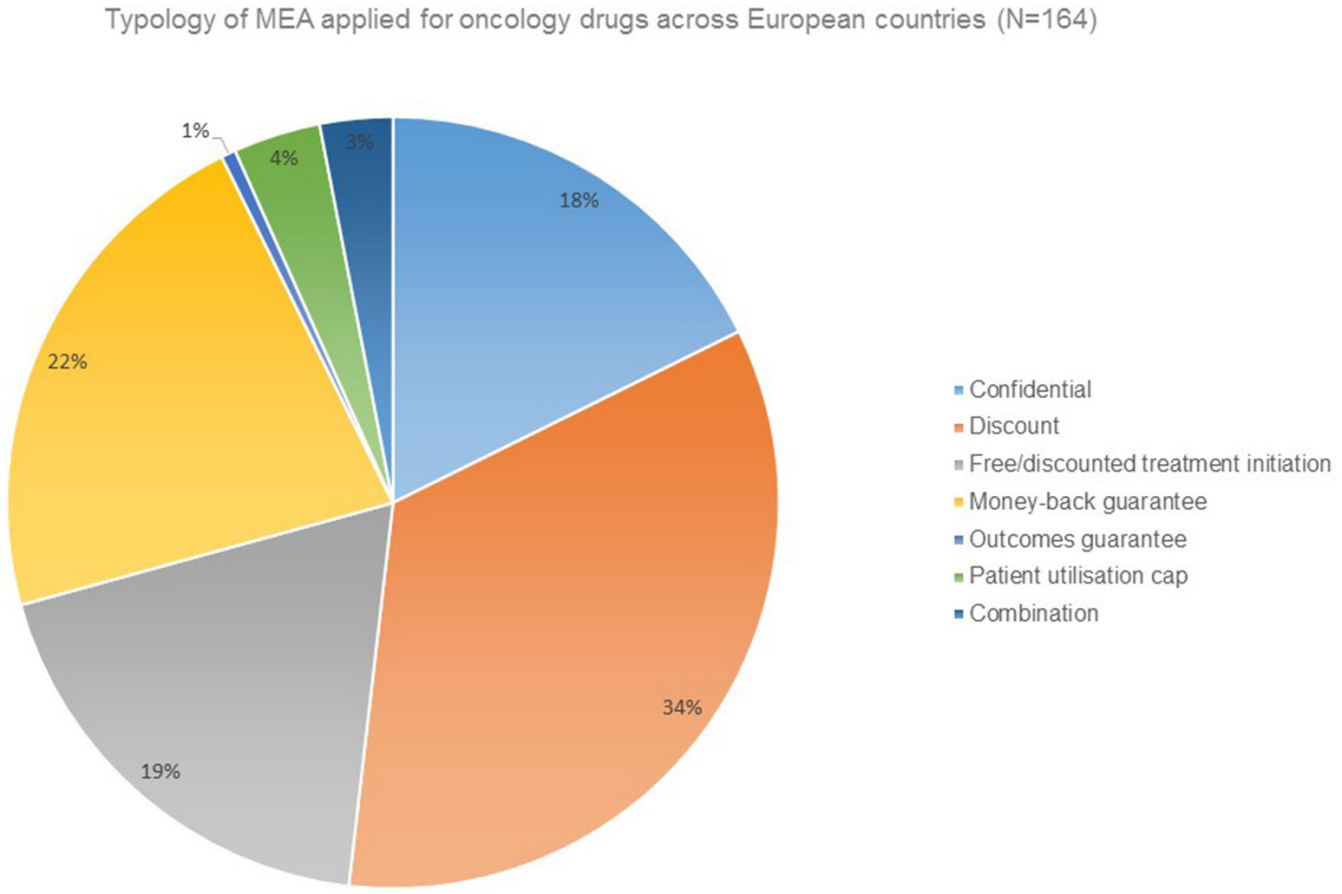
**Typology of MEA applied for oncology drugs across European countries (***N*** = 164)**.

Financial agreements in the form of discounts are the prevailing form of MEA applied for oncology drugs (Figure 1).

In England, Wales, and Scotland, the PPRS specifies that MEA should be clinically robust, clinically plausible, appropriate, and operationally manageable for the National Health Service (NHS) without unduly complex monitoring, disproportionate additional costs and bureaucracy. Therefore, simple discount schemes take the lead, although the option for complex schemes remains available. Complex schemes include all other types of MEA such as rebates (which involve a separate transaction whereas a discount is applied directly to original invoices), stock supplied at zero cost (free stock), dose capping (agreement of manufacturers and payers on a pre-determined level of consumption, with manufacturers covering any additional costs incurred beyond this limit), and outcome-based schemes called response schemes. Scotland considers whether a MEA applied by England is feasible in Scotland, given infrastructure differences between England and Scotland which can influence the feasibility of a particular scheme, for example different IT capability. Furthermore, differences in supply chain of medicines can have an impact on the feasibility, illustrated by the involvement of community pharmacies in the supply of specialty medicines in Scotland, which is not the case in England.

Also in Czech Republic, the majority of MEA (13/19) involve a simple confidential discount, referred as price declaration. MEA are applied to meet the willingness to pay (WTP), which is considered to be three times the GDP of the country.

For Belgium, no details on the characteristics of the MEA are available due to confidentially. The annual report of the National Institute of Health and Disability Insurance (NIHDI) however states that, in the majority of MEA the health insurance will initially bear the costs of drugs and the MAH will rebate an agreed amount of money to the NIHDI after a specified period of time. The rebate can be determined in function of the turnover of the MAH or the rebate can be based on the performance of the drug^4^.

In Sweden, all MEA for oncology drugs involve a patient utilization cap. Therefore, a national registry funded by the Government and set up by the National Board of Health and Welfare delivers data on volume and use of pharmaceuticals, based on the input of pharmacists. Registries are also available at the county councils, although its use for MEA is complicated due to heterogeneity across the councils and therefore, patient utilization caps are declined for in-patient medicines in the system that is in place in 2016.

In France, a study conducted by European Medicines Information Network (EMINet) reported that a price-volume contract is applied for an oncology drug between 2010 and 2015 with the aim to control budget, but its name is not disclosed due to confidentiality (Espin et al., 2011).

In the Netherlands, financial agreements have been applied for extramural care since 2012 in case of an unfavorable cost-benefit ratio or when the budget impact is more than average and the situation of the market can not address the high price or high expenditures, for example due to a lack of competitor drugs. In 2015, the application of financial agreements was extended to inpatient drugs as well. During a horizon scan, the cost and volume consequences of products that are expected to enter the market in the next year are evaluated. When the costs are too high and it is unlikely that these can be addressed by the market situation or negotiations held under the responsibility of the hospitals themselves, the drugs are “locked” from entering the market until negotiation of an agreement. Financial agreements imply that the company will rebate a certain amount to the Health Insurers, over a specified period of time. The rebate can be organized in different ways, such as price/volume agreements or discounts. The rebates are confidential and organized by a trusted third party, who is responsible for administrative aspects of the rebate but not involved in the negotiation. In April 2015, mutual negotiations between the Netherlands and Belgium, extended by Luxembourg in September 2015 and by Austria in June 2016, were announced but experiences were not available at the moment of publication^5^,^6^.

### Performance-based agreements

In Italy, a majority of MEA are performance-based to decide on a refund for non-responders (35/63) at patient-individual level. Six percent have both performance-based as well as financially based aspects (4/63). Standard monitoring registries are applied independently from MEA, and can be employed to monitor patient response in the context of performance-based agreements. These registries are web-based tools organized by the Italian Medicines Agency (AIFA) to share clinical information and safety data between regulators, clinicians and pharmacists. Registries are based on IT facilities that receive financial support from pharmaceutical companies.

Also in the Netherlands, several schemes are set-up since 2008, in the context of the policy measure for expensive medicines and OMP, but the Health Care Institute (Zorginstituut) experienced that data gathered over the last years only poorly address the uncertainties that were left at the moment that reimbursement was filed in the Netherlands. The quality of the data collected within such schemes depended on the engagement of clinicians, who have little incentive to devote resources and time to data collection. The validity of the data collected within such schemes was often affected by scientific evolutions, new product launches in the market and changing clinical practice that result thereof, especially in dynamic domains such as oncology. Therefore, appropriate use is preferred instead of follow-up research. Examples on measures for appropriate use involve designation of expertise centers and the definition of start and stop criteria for use of the medicine. In 2016, the Health Care Institute (Zorginstituut) was evaluating the feasibility and usefulness of a re-evaluation for each dossier and the manufacturers will be informed about the decision.

### Duration of the agreements

In England and Wales, the lifetime of the relevant guidance from the National Institute of Health and Care Excellence (NICE) determines the duration of the MEA, but the exact agreement needs to be clear and conditions on MEA's termination need to be specified. In case of new indications or changes in the scheme type, a new MEA submission is required.

In Scotland, MEA are by rule applied for 5 years, unless there is a price decrease. After 5 years, the company can indicate to continue or stop the MEA however the product should continue to be available at the price agreed within the MEA or a lower list price.

In Italy, decision on price setting and reimbursement are usually re-evaluated after 24 months, but the duration of MEA can be adjusted on a case-by-case basis. MEA can be extended at time of re-evaluation or the content can change, illustrated by Adcetris® where a performance-based scheme was switched to a financial agreement in July 2014 for both Hodgkin and Non-Hodgkin lymphoma.

In the Netherlands, the duration of a financial agreement is dependent on the product and on future products that are expected to enter the market. It is continuously evaluated whether the price cannot be subjected to market forces such as competition. The dynamics in the oncology domain, combination of treatments and multiple indications for certain drugs make the oncology domain complex as for some indications competition is possible and for others not.

Also in the Czech Republic, the market is continuously evaluated. The duration of a MEA is usually 3 years and afterwards, new information gained from registries, experience in other countries or published studies about effectiveness or safety will be considered during re-evaluation.

In Belgium, the duration of a MEA is case dependent and varies between 2 and 3 years. The majority of MEA for which the end date has passed, were extended. Furthermore, MEA continue despite the introduction of competitor treatments, which are also subjected to a MEA at their turn (Supplementary Information).

## Discussion

To the best of the authors' knowledge, this study is the first to provide a comprehensive overview of the current practice of MEA applied for oncology drugs (including drugs that intend to treat solid tumors as well as hematological malignancies) in a set of European countries.

The application of MEA is heterogeneous across countries (Ferrario and Kanavos, 2015). First, the variety in health care systems and economic affluence of individual European countries leads to heterogeneous price setting and reimbursement practices across different countries (Garattini and Bertele, 2002; Edlin et al., 2014). Second, contextual differences with regard to data collection infrastructure and supply chain of specialty medicines influence the feasibility to apply the different types of MEA in a certain country. Contextual factors can differ between countries but also within countries dependent on regions (i.e., Sweden) or territories (UK). Despite the fact that the application of MEA is heterogenous across European countries, MEA contribute to accessibility at the level of individual countries by allowing patient access to drugs that would not be reimbursed otherwise.

Our study shows that as of 2016, financial based agreements in the form of direct or indirect price reductions were the most common form of MEA applied for oncology drugs in the countries included in this study. The same observation is reported by studies that consider MEA across all disease domains (Vogler et al., 2012; Morel et al., 2013; Executive Insight Health Care Consultants, 2016). Studies conducted before 2015, however, show that performance-based agreements are commonly applied in Sweden and the Netherlands (Morel et al., 2013; Ferrario and Kanavos, 2015; van de Vooren et al., 2015). As payers were struggling to define and measure the outcome, they increasingly refrained from this type of MEA. The poor relationship between short term surrogate outcomes and hard endpoints is, in general, a major limitation of performance-based schemes in which reimbursement is coupled to performance of the drug in either a patient population (coverage with evidence development) or at patient individual level (Ferrario and Kanavos, 2015). Furthermore, the value of information will typically be lower than the cost of data collection (Edlin et al., 2014).

Rapidly evolving markets, such as present for oncology, require a dynamic approach for the value of drugs where mechanisms for market access can adapt to new competitors and comparators are introduced. Data collection at the level of a patient group, such as applied by coverage with evidence development, is a rigid system in which conditions for data collection are fixed over longer periods of time. Due to limited flexibility to adapt data requirements in function of new evolutions in the market, the relevance of data collected is often disputed. Nevertheless, a study conducted by Toumi et al. showed that coverage with evidence agreements can provide answers about uncertainties in real world effectiveness by using patient relevant outcomes (Toumi et al., 2017). Although performance-based agreements at patient individual level can allow more flexibility to adapt to changes in the market over time, rigid outcomes based on surrogate end-points, are applied in pay for performance agreements (Toumi et al., 2017).

Experience with performance-based schemes on individual patient level has meanwhile been made for more than 10 years in Italy. Payback by the MAH of treatment for non-responding patients faced challenges due to inefficiency of health care centers who have to collect the data and claim payback^4^. Additionally, interruptions of treatment due to reasons other than what is agreed in the context of the scheme can lead to dispute about the payback of treatments (Navarria et al., 2015). Success fees, also described as outcomes guarantee in the literature (Morel et al., 2013), apply a retrospective payment by the payer for patients who have benefited from a treatment^4^. In our study, outcome guarantees applied for oncology drugs were only observed in one single case in Scotland. Outcomes guarantees linked to high thresholds for response can increases the likelihood that the drug will be paid by the MAH, limiting the costs for the public payer, especially for high cost oncology drugs. When the agreement expires, the content of the agreement will be considered during the negotiation fur future reimbursement. The incentive to set high thresholds for response can limit the use of drugs for certain types of patients, and despite the benefits for the public health care budget, patient advocates can perceive this as a threat. On the other hand, drugs are supplied for free within the context of an outcomes guarantee or similar schemes that apply conditional treatment continuation, and therefore there is the risk that they are used beyond the most beneficial patient population. Defining objective, clinically meaningful outcomes that are measurable within restricted timeframes and available IT infrastructure will be challenging.

Financial agreements offer financial security for payers at a lower cost of data collection, however, the administrative workload can still place a burden on health care systems. Although the hurdles in price setting and reimbursement are clearly composed by clinical uncertainties (relative efficacy or effectiveness), financial uncertainties (budget impact) or a combination of both (cost-effectiveness), our study showed that simple discount schemes take the lead, despite the fact that this type of agreement shows the least opportunities for sharing the risk (i.e., cost of uncertainties) between the payer and the MAH. External reference price setting (i.e., price setting based on the price in other countries) in Europe is one of the economic drivers behind the application of MEA. MEA allow MAHs to differentiate prices between countries in a confidential manner, impeding the effect of a price decrease in reference countries on the price of other countries (Leopold et al., 2012). MEA and other confidential price reductions flaw the system of external reference price setting as the list price does not reflect the real price covered by the payers. Furthermore, focus on financial impact instead of drug value can urge MAHs to anticipate price decreases and consequently, payers will distrust the price proposed by MAHs (Vogler et al., 2012; Executive Insight Health Care Consultants, 2016; Pauwels, 2016).

Oncology drugs are characterized by multiple indications with more than 50% of major cancer medicines marketed for multiple indications in 2014 (Mestre-Ferrandiz et al., 2015)^7^. Even within a certain type of cancer, the benefits of the drug will differ dependending on the stage of the disease and the risks that will be considered acceptable will be higher for advanced stage disease compared to first line settings. This study showed that in a limited number of countries, e.g., Belgium and Italy, MEA allow to distinct the price across indications, despite a single list price that is valid for all indications for which the drug is marketed. In UK, the launch of new indications for a product subjected to a MEA requires that the MEA is reconsidered, taking into account the full set of indication. In addition to benefit that are indication specific, it is unlikely that the uncertainties can be generalized across different indications for a given drugs and therefore, MEA can only be based on evidence when specific indications are considered. Due to the dynamics of the oncology market, fixed contract durations can only be applied when the launch of a specific drug in new indications, or the launch of other drugs in the same indication, is taken into account based on horizon scanning. While the application of indication specific MEA can provide incentives to the pharmaceutical industry to broaden indication of oncology drugs, even when the benefits are lower compared to the initial indication, applying a single MEA to different indications for the same product can discourage industry to apply for reimbursement in new indications as this can induce additional discounts or rebates within a MEA (Pauwels et al., 2016). Although the latter case in theory can limit the reimbursement of the product to those indications where the highest value is perceived, it can jeopardize accessibility to oncology drugs for patients in high need or it can induce off-label use of the product with unexpected costs for public health care systems as a consequence.

A large number of the identified MEA was extended after expiration. On the one hand this can be due to the lack of answers toward the uncertainties that were formulated. On the other hand, MEA for different drugs that intent to treat the same indication were observed within some countries during this study and are likely to maintain the existence of a contract over time. When a drug for which reimbursement is applied, is compared to alternatives with a confidential price, the new application is likely to result in a MEA as well. Due to extensive prolongation of MEA, the MEA can persist through the lifecycle of the product, and the question on how to deal with generic competition for products that have a confidential price due to a MEA will arrive soon.

This study suffers from several limitations. No complete data set could be obtained due to confidentiality in some countries. Unavailability of data was partially compensated by a descriptive overview. Furthermore, caution is needed with regard to the comparability of the application and regulatory framework for MEA in different countries that each have their own legislation with regard to health policy. This provided however a broad range of experiences from which countries can learn.

## Conclusions

MEA appear to be a common policy instrument for public payer given the complexities of price setting and reimbursement of drugs. Given the pressure on authorities to ensure earlier market access of drugs, as well as new challenges due to new therapeutic mechanisms such as immune oncology, MEA are likely to stay in the future oncology drug market. The experience from the past however shows that in order to exploit the benefits of MEA related to accessibility for patients, affordability for society and profitability by pharmaceutical companies, the dynamics of the oncology market need to be taken into account when MEA are set up.

## Author contributions

All authors contributed to the conception and design of the study. KP was involved in data collection and data analysis. All authors were involved in interpretation of the results, and contributed to drafting and revising the manuscript. All authors approved the final version of the manuscript.

## Funding

This study was funded by Research Foundation Flanders and Flanders Innovation and Entrepreneurship.

### Conflict of interest statement

The authors declare that the research was conducted in the absence of any commercial or financial relationships that could be construed as a potential conflict of interest.

## Footnotes

^1^European Medicines Agency. 2015. 18-2-2017. Ref Type: Online Source.

^2^Wilkings. Patient access for oncology drugs. 2012. Ref Type: Internet Communication.

^3^French Healthcare products pricing committee. 2015/2015 Annual Report. 2016.

^4^RIZIV/INAMI. M.O.R.S.E semesterieel rapport 2014.(2). 2012. 20-1-2013. Ref Type: Online Source.

^5^Ministre des Affaires sociales et de la Santé publique. The Grand Duchy of Luxemburg Joins Belgium-Netherlands Initiative on Orphan Drugs. 24-9-2015. Ref Type: Online Source.

^6^Bundesministerium fur gezundheit. Österreich tritt Benelux-Kooperation für Arzneimittelpolitik bei. 17-6-2016. Ref Type: Online Source.

^7^IMS Institute for healthcare informatics. Developments in Cancer Treatments, Market dynamics, Patient Access and Value. 2015. 29-2-2016. Ref Type: Online Source.

## References

[B1] Abboud C. Berman E. Cohen A. Cortes J. DeAngelo D. Deininger M. (2013). The price of drugs for chronic myeloid leukemia (CML) is a reflection of the unsustainable prices of cancer drugs: from the perspective of a large group of CML experts. Blood. 121, 4439–4442. 10.1182/blood-2013-03-490003 23620577 PMC4190613

[B2] Carlson J. J. Garrison L. P. Sullivan S. D. (2009). Paying for outcomes: innovative coverage and reimbursement schemes for pharmaceuticals. J. Manag. Care Pharm. 15, 683–687. 10.18553/jmcp.2009.15.8.683 19803557 PMC10438050

[B3] Edlin R. Hall P. Wallner K. McCabe C. (2014). Sharing risk between payer and provider by leasing health technologies: an affordable and effective reimbursement strategy for innovative technologies? Value Health 17, 438–444. 10.1016/j.jval.2014.01.010 24969005

[B4] Eichler H. G. Baird L. G. Barker R. BlØechl-Daum B. Borlum-Kristensen F. Brown J.. (2015). From adaptive licensing to adaptive pathways: delivering a flexible life-span approach to bring new drugs to patients. Clin. Pharmacol. Ther. 97, 234–246. 10.1002/cpt.59 25669457 PMC6706805

[B5] Espin J. Rovira J. Garcia L. (2011). Experiences and Impact of European Risk-Sharing Schemes Focusing on Oncology Medicines. European Commission.

[B6] Executive Insight Health Care Consultants (2016). Innovative Contracting: A Review.

[B7] Ferrario A. Kanavos P. (2015). Dealing with uncertainty and high prices of new medicines: a comparative analysis of the use of managed entry agreements in Belgium, England, the Netherlands and Sweden. Soc. Sci. Med. 124, 39–47. 10.1016/j.socscimed.2014.11.003 25461860

[B8] Fojo T. Grady C. (2009). How much is life worth: cetuximab, non-small cell lung cancer, and the $440 billion question. J. Natl. Cancer Inst. 101, 1044–1048. 10.1093/jnci/djp177 19564563 PMC2724853

[B9] Fojo T. Lo A. W. (2016). Price, value, and the cost of cancer drugs. Lancet Oncol. 17, 3–5. 10.1016/S1470-2045(15)00564-1 26758749

[B10] Garattini S. Bertele V. (2002). Efficacy, safety, and cost of new anticancer drugs. BMJ. 325, 269–271. 10.1136/bmj.325.7358.269 12153927 PMC1123779

[B11] Gozzo L. Navarria A. Drago V. Longo L. Mansueto S. Pignataro G.. (2016). Linking the price of cancer drug treatments to their clinical value. Clin. Drug Investig. 36, 579–589. 10.1007/s40261-016-0403-1 27153824

[B12] Jönsson B. Hofmarcher T. Lindgren P. Wilking N. (2016). The cost and burden of cancer in the European Union 1995-2014. Eur. J. Cancer 66, 162–170. 10.1016/j.ejca.2016.06.022 27589247

[B13] Leopold C. Vogler S. Mantel-Teeuwisse A. K. de Joncheere K. Leufkens H. G. Laing R. (2012). Differences in external price referencing in Europe: a descriptive overview. Health Policy 104, 50–60. 10.1016/j.healthpol.2011.09.008 22014843

[B14] Mestre-Ferrandiz J. Towse A. Dellamano R. Pistolllato M. (2015). Multi-Indication Pricing: Pros, Cons and Applicability to the UK.

[B15] Morel T. Arickx F. Befrits G. Siviero P. van der Meijden C. Xoxi E.. (2013). Reconciling uncertainty of costs and outcomes with the need for access to orphan medicinal products: a comparative study of managed entry agreements across seven European countries. Orphanet J. Rare Dis. 8:198. 10.1186/1750-1172-8-198 24365263 PMC3882782

[B16] Navarria A. Drago V. Gozzo L. Longo L. Mansueto S. Pignataro G.. (2015). Do the current performance-based schemes in Italy really work? “Success fee”: a novel measure for cost-containment of drug expenditure. Value Health. 18, 131–136. 10.1016/j.jval.2014.09.007 25595244

[B17] Pauwels K. (2016). Financial based agreements and performance based agreements: the Belgian experience. J. Pharm. Policy Practi. 8(Suppl 1): O1 10.1186/2052-3211-8-S1-O1

[B18] Pauwels K. Huys I. Casteels M. De Nys K. Simoens S. (2014). Market access of cancer drugs in European countries: improving resource allocation. Target. Oncol. 9, 95–110. 10.1007/s11523-013-0301-x 24243526

[B19] Pauwels K. Huys I. Casteels M. Simoens S. (2016). Industry perspectives on market access of innovative drugs: the relevance for oncology drugs. Front. Pharmacol. 7:144. 10.3389/fphar.2016.00144 27313529 PMC4887481

[B20] Toumi M. Jaroslawski S. Sawada T. Kornfeld Å. (2017). The use of surrogate and patient-relevant endpoints in outcomes-based market access agreements: current debate. Appl. Health Econ. Health Policy 15, 5–11. 10.1007/s40258-016-0274-x 27581118

[B21] van de Vooren K. Curto A. Freemantle N. Garattini L. (2015). Market-access agreements for anti-cancer drugs. J. R. Soc. Med. 108, 166–170. 10.1177/0141076814559626 25488094 PMC4484207

[B22] Vogler S. Vitry A. Babar Z. U. (2015). Cancer drugs in 16 European countries, Australia, and New Zealand: a cross-country price comparison study. Lancet Oncol. 17, 39–47. 10.1016/S1470-2045(15)00449-0 26670089

[B23] Vogler S. Zimmermann N. Habl C. Piessnegger J. Bucsics A. (2012). Discounts and rebates granted to public payers for medicines in European countries. South. Med. Rev. 5, 38–46. 23093898 PMC3471187

