# Supplementary material for: Managed Entry Agreements for Oncology Drugs: Lessons from the European Experience to Inform the Future
Source: Front Pharmacol. 2017 Apr 4;8:171. doi: 10.3389/fphar.2017.00171 (PMC5378787; doi:10.3389/fphar.2017.00171)
Supplement: Supplementary file 1 [file DataSheet1.pdf]

Country	Product	Substance	Company	ATC-code	OMP	Start date	End date	Date of Prolongation	Indication	Financial based (F) or performance-based (P) agreement	Typology
Belgium	Adcetris	Brentuximab	Takeda Belgium	L01XC12	1	Nov 2014	Nov 2016		Relapsed or refractory CD30+ Hodgkin lymphoma (HL), following autologous stem cell transplant (ASCT) or following at least two prior therapies when ASCT or multi-agent chemotherapy is not a treatment option+relapsed or refractory systemic sALCL	Confidential	Confidential
Belgium	Avastin	Bevacizumab	Roche	L01XC07	0	Sept 2014	March 2017		Colorectal cancer, second line	Confidential	Confidential
Belgium	Avastin	Bevacizumab	Roche	L01XC07	0	July 2015	March 2017		Platinum-resistant recurrent epithelial ovarian cancer, in combination with paclitaxel, topotecan or doxorubicin	Confidential	Confidential
Belgium	Avastin	Bevacizumab	Roche	L01XC07	0	March 2014	March 2017		Ovarian cancer, 1st line and 2nd line	Confidential	Confidential
Belgium	Caprelsa	Vandetanib	AstraZeneca	L01XE12	0	May 2013	May 2016		Medullar thyroid gland cancer	Confidential	Confidential
Belgium	Dacogen	Decitabine	Janssen-cilag	L01BC08	1	Dec 2013	Dec 2016		Newly diagnosed de novo or secondary acute myeloid leukaemia (AML) in treatment of adults older than 65years, who are not candidates for standard induction chemotherapy	Confidential	Confidential
Belgium	Erivedge	Vismodegib	Roche	L01XX43	0	Oct 2014	Oct 2017		Locally advanced basal cell carcinoma, inappropriate for surgery or radiotherapy and symptomatic metastatic basal cell carcinoma	Confidential	Confidential
Belgium	Gazyvaro	Obinutuzumab	Roche	L01XC15	1	Oct 2015	Oct 2018		Chronic lymphocytic leukaemia, first line, in combination with chlorambucil, in patients with comorbidities making them	Confidential	Confidential

									unsuitable for full-dose fludarabine based therapy		
Belgium	Halaven	Eribulin	Eisai europe	L01XX45	0	Oct 2013	Oct 2016		Locally advanced or metastatic breast cancer after at least two chemotherapeutic regimens for advanced disease	Confidential	Confidential
Belgium	Imbruvica	Ibrutinib	Janssen-cilag	L01XE27	1	Aug 2015	Aug 2018		Relapsed or refractory mantle cell lymphoma + chronic lymphocytic leukaemia, at least 1 prior therapy or in 1st line in presence of 17p deletion or TP53 mutation in patients unsuitable for chemo-immunotherapy	Confidential	Confidential
Belgium	Imnovid	Pomalidomide	Celgene	L04AX06	1	Sept 2014	Sept 2017		Relapsed and refractory multiple myeloma, after at least two prior treatment regimens including lenalidomide and bortezomib, and a prior treatment with an alkylant, and have demonstrated disease progression on the last therapy	Confidential	Confidential
Belgium	Jevtana	Cabazitaxel	Sanofi-aventis Belgium	L01CD04	0	May 2012	May 2013	May 2014 + May 2015 + May 2016	Hormone refractory metastatic prostate cancer, after treatment with a docetaxel-containing regimen	Confidential	Confidential
Belgium	Kadcyla	Ado-trastuzumab Emtansine	Roche	L01XC14	0	Dec 2014	Dec 2017		HER2-positive, unresectable locally advanced or metastatic breast cancer, after trastuzumab and a taxane, separately or in combination	Confidential	Confidential
Belgium	Lynparza	Olaparib	AstraZeneca	L01XX47	1	Dec 2015	Dec 2017		Platinum-sensitive relapsed BRCA-mutated (germline and/or somatic) high grade serous epithelial ovarian, fallopian tube, or primary peritoneal cancer who are in response (complete response or partial response) to platinum-based chemotherapy.	Confidential	Confidential
Belgium	Mabthera	Rituximab	Roche	L01XC02	0	Dec 2014	Jan 2016		Chronic lymphocytic leukemia, previously untreated, relapsed or refractory, in combination with chemotherapy	Confidential	Confidential

Belgium	Perjeta	Pertuzumab	Roche	L01XC13	0	June 2014	June 2017		HER2-positive metastatic or locally recurrent unresectable breast cancer, not received previous anti-HER2 therapy or chemotherapy for their metastatic disease	Confidential	Confidential
Belgium	Revlimid	Lenalidomide	Celgene	L04AX04	1	Oct 2014	Oct 2017		Transfusion-dependent anaemia due to low- or intermediate-1-risk myelodysplastic syndromes associated with an isolated deletion 5q cytogenetic abnormality, when other therapeutic options are insufficient or inadequate	Confidential	Confidential
Belgium	Sprycel	Dasatinib	Bristol-myers squibb Belgium	L01XE06	1	Dec 2011	Dec 2014	Dec 2015	Newly diagnosed Philadelphia chromosome-positive (Ph+) chronic myelogenous leukemia (CML) in chronic phase	Confidential	Confidential
Belgium	Torisel	Temsirolimus	Pfizer	L01XE09	0	Sept 2011	Sept 2014		Relapsed and/or refractory mantle cell lymphoma	Confidential	Confidential
Belgium	Xalkori	Crizotinib	Pfizer	L01XE16	0	Aug 2013	Aug 2016		Previously treated ALK-positive advanced non-small cell lung cancer	Confidential	Confidential
Belgium	Xtandi	Enzalutamide	Astellas	L02BB04	0	Dec 2014	Dec 2016		Metastatic castration-resistant prostate cancer, progressed on or after docetaxel therapy	Confidential	Confidential
Belgium	Xtandi	Enzalutamide	Astellas Pharma	L02BB04	0	Oct 2015	Dec 2016		Metastatic castration resistant prostate cancer in pre chemo (docetaxel) setting	Confidential	Confidential
Belgium	Yervoy	Ipilimumab	Bristol-Meyers Squibb Belgium	L01XC11	0	June 2015	June 2016		Treatment of advanced (unresectable or metastatic) melanoma in adults in first line	Confidential	Confidential
Belgium	Yervoy	Ipilimumab	Bristol-Myers Squibb Belgium	L01XC11	0	June 2012	June 2016		Treatment of advanced (unresectable or metastatic) melanoma after prior therapy	Confidential	Confidential
Belgium	Zaltrap	Aflibercept	Sanofi-Aventis Belgium	L01XX44	0	Nov 2014	Nov 2017		Metastatic colorectal cancer that is resistant to or has progressed after an oxaliplatin-containing regimen, in combination with irinotecan/5-	Confidential	Confidential

									fluorouracil/folinic acid (FOLFIRI) chemotherapy		
Belgium	Zelboraf	Vemurafenib	Roche	L01XE15	0	April 2013	July 2014	May 2015	Treatment of non-resectable or metastatique melanoma with BRAF V600 mutation	Confidential	Confidential
Belgium	Zytiga	Abiraterone Acetate	Janssen-cilag	L02BX03	0	Aug 2012	Aug 2015	Aug 2017	metastatic castration resistant prostate cancer progressed on or after a docetaxel-based chemotherapy regimen, in combination with prednisone or prednisolone	Confidential	Confidential
Belgium	Zytiga	Abiraterone acetate	Janssen-Cilag	L02BX03	0	Feb 2014	Aug 2016		Metastatic castration resistant prostate cancer, asymptomatic or mildly symptomatic after failure of androgen deprivation therapy when chemotherapy is not yet clinically indicated	Confidential	Confidential
England	Arzerra	Ofatumumab	Novartis	L01XC10	1	June 2015			Chronic lymfocytic leukemia, first line, in combination with chlorambucil	F	Discount
England	Gazyvaro	Obinutuzumab	Roche	L01XC15	1	June 2015			Chronic lymphocytic leukemia, first line	F	Discount
England	Giotrif	Afatinib	Boehringer Ingelheim	L01XE13	0	April 2014			Locally advanced or metastatic non-small cell lung cancer	F	Discount
England	Inlyta	Axitinib	Pfizer	L01XE17	0	Feb 2015			Advanced renal cell carcinoma after failure of prior systemic treatment	F	Discount
England	Iressa	Gefitinib	AstraZeneca	L01XE02	0	July 2010			Locally advanced or metastatic non-small-cell lung cancer, first line	F	Free/discounted treatment initiation
England	Keytruda	Pembrolizumab	Merck Sharp & Dohme	L01XC18	0	Nov 2015			Advanced melanoma not previously treated with ipilimumab	F	Discount

England	Keytruda	Pembrolizumab	Merck Sharp & Dohme	L01XC18	0	Oct 2015	Advanced melanoma after disease progression with ipilimumab	F	Discount
England	Mepact	Mifamurtide	Takeda	L03AX01	1	Oct 2011	High grade, non-metastatic osteosarcoma after surgical resection	F	Discount
England	Pixuvri	Pixantrone	CTI life sciences	L01DB11	0	Feb 2014	Relapsed or refractory aggressive non-hodgkin lymfoma	F	Discount
England	Revlimid	Lenalidomide	Celgene	L04AX04	1	Sept 2014	Myelodysplastic syndromes with an isolated deletion 5q cytogenetic abnormality	F	Patient utilisation cap
England	Sutent	Sunitinib	Pfizer	L01XE04	0	March 2009	Unresectable or metastatic malignant gastro-intestinal tumor (GIST)	F	Free/discounted treatment initiation
England	Sutent	Sunitinib	Pfizer	L01XE04	0	March 2009	First line treatment of advanced/metastatic renal cell carcinoma	F	Free/discounted treatment initiation
England	Tafinlar	Dabrafenib	GSK	L01XE23	0	Oct 2014	Unresectable or metastatic melanoma with BRAFV600 mutation	F	Discount
England	Tarceva	Erlotinib	Roche	L01XE03	0	Dec 2015	Non-small-cell lung cancer that has progressed after prior chemotherapy	F	Discount
England	Tarceva	Erlotinib	Roche	L01XE03	0	June 2012	Locally advanced and metastatic positive non- small-cell lung cancer with EGFR-TK mutation, first line	F	Discount
England	Tasigna	Nilotinib	Novartis	L01XE08	1	Dec 2015	Chronic myeloid leukemia, first line	F	Discount
England	Tasigna	Nilotinib	Novartis	L01XE08	1	June 2012	Chronic myeloid leukemia resistant to imatinib	F	Discount

England	Vargatef	nintedanib	Boehringer Ingelheim	L01XE31	0	July 2015	Previsouly treated locally advanced metastatic or locally recurrent non-small cell lung cancer	F	Discount
England	Vidaza	Azacitidine	Celgene	L01BC07	1	March 2011	Myelodysplastic syndrome, chronic myelodysplastic leukemia and acute myeloid leukemia	F	Discount
England	Votrient	Pazopanib	GSK	L01XE11	0	Feb 2011	Advanced renal cell carcinoma, first line	F	Discount
England	Yervoy	Ipilimumab	Bristol-Myers Squibb	L01XC11	0	July 2014	Advanced (unresectable or metastatic) melanoma, first line	F	Discount
England	Yervoy	Ipilimumab	Bristol-Myers Squibb	L01XC11	0	July 2014	Advanced (unresectable or metastatic) melanoma, second line	F	Discount
England	Yondelis	Trabectedin	PharmaMar	L01XE01	1	2010	Advanced tissue sarcoma	F	Patient utilisation cap
England	Zelboraf	Vemurafenib	Roche	L01XE15	0	Dec 2012	Locally advanced (unresectable or metastatic) malignant myeloma, positive for BRAF V600 mutation	F	Discount
England & Wales	Erbitux	Cetuximab	Merck	L01XC06	0	Sept 2009	Metastatic colorectal cancer, first line	F	Discount
England & Wales	Revlimid	Lenalidomide	Celgene	L04AX04	1	June 2009	Multiple myeloma after at least two other treatments	F	Patient utilisation cap
England & Wales	Xtandi	Enzalutamide	Astellas Pharma	L02BB04	0	July 2014	Metastatic hormone relapsed prostate cancer, progressed during or after docetaxel	F	Discount
England & Wales	Zytiga	Abiraterone	Janssen-cilag	L02BX03	0	June 2012	Castration resistant metastatic prostate cancer previously treated with docetaxel-containing regimen	F	Discount

Italy	Abraxane	Paclitaxel-albumine	Celgene	L01CD01	0	Feb 2015			Pancreatic neoplasms, in association with gemcitabine	P	Money-back guarantee
Italy	Adcetris	Brentuximab	Takeda	L01XC13	1	Nov 2012	July 2014		Non-Hodgkin lymphoma	P	Money-back guarantee
Italy	Adcetris	Brentuximab	Takeda	L01XC14	1	Nov 2012	July 2014		Hodgkin lymphoma	P	Money-back guarantee
Italy	Afinitor	Everolimus	Novartis	L04AA18	0	July 2013			Breast neoplasms	P	Money-back guarantee
Italy	Afinitor	Everolimus	Novartis	L04AA18	0	June 2010			Renal cell carcinoma	P	Money-back guarantee
Italy	Afinitor	Everolimus	Novartis	L04AA18	0	Dec 2012			Pancreatic neoplasms	P	Money-back guarantee
Italy	Alimta	Pemetrexed	Elli Lilly	L01BA04	0	May 2009		July 2014	Locally advanced or metastatic non-small cell lung cancer, in combination with cisplatina for first line, maintance treatment or second line treatment	P	Money-back guarantee
Italy	Avastin	Bevacizumab	Roche	L01XC07	0	Oct 2005		July 2014	Colorectal carcinoma 2nd line	P	Money-back guarantee
Italy	Avastin	Bevacizumab	Roche	L01XC07	0	April 2014			Platina sensitive epithelial ovarian neoplasms, previously treated with bevacizumab or other products targeting VEGFR, in combination with carboplatin and gemcitabine, 1ste line and 2nd line	P	Money-back guarantee
Italy	Avastin	Bevacizumab	Roche	L01XC07	0	July 2008	July 2014		Breast neoplasms	P+F	Dose capping + money-back guarantee

Italy	Avastin	Bevacizumab	Roche	L01XC07	0	July 2008	July 2014		Renal cell carcinoma	P+F	Dose capping + money-back guarantee
Italy	Avastin	Bevacizumab	Roche	L01XC07	0	July 2008	July 2014		Non-small-cell Lung Carcinoma	P+F	Dose capping + money-back guarantee
Italy	Bosulif	Bosutinib	Pfizer	L01XE14	1	Oct 2014			Philadelphia-chromosome positive chronic myeloid leukemia (Ph + CML) in chronic phase, acceleration phase and blast phase, previously treated with one or more tyrosine kinase inhibitors and not appropriate for treatment with imatinib, nilotinib and dasatinib.	P	Money-back guarantee
Italy	Erbitux	Cetuximab	Eli lilly	L01XC06	0	Dec 2010			Head and neck neoplasms	P	Money-back guarantee
Italy	Erbitux	Cetuximab	Eli Lilly	L01XC06	0	Oct 2008		Oct 2014	Metastatic colorectal carcinoma with expression of EGFR and without RAS mutations. In combination with irinotecan, FOLFOX or monotherapie when intolerant or unresponsive for irinotecan	P	Money-back guarantee
Italy	Giotrif	Afatinib	Boehringer Ingelheim	L01XE13	0	Dec 2014			Non-small-cell lung carcinoma	P	Money-back guarantee
Italy	Halaven	Erbulin	Eisai	L01XX45	0	Jan 2012		May 2015	Locally advanced breast cancer after at least two treatments with anthracycline and taxanes	P	Money-back guarantee
Italy	Herceptin	Trastuzumab	Genentech	L01XC03	0	Jan 2011			Stomach neoplasms	P	Money-back guarantee
Italy	Iclusig	Ponatinib	Ariad	L01XE24	1	Dec 2014			Lymphoid and myeloid leukemia	P	Money-back guarantee

Italy	Inlyta	Axitinib	Pfizer	L01XE17	0	Jan 2014			Renal cell carcinoma after failure of previous treatment with sunitib or a cytokine	P	Money-back guarantee
Italy	Iressa	Gefitinib	AstraZeneca	L01XE02	0	June 2010			Non-small-cell lung carcinoma	P	Money-back guarantee
Italy	Javlor	Vinflunine	Pierre Fabre	L01CA05	0	Jan 2011			Monotherapy for urothelial advanced or metastatic cell carcinoma after platinum consisting therapy	P	Money-back guarantee
Italy	Kadcyla	Trastuzumab emtansine	Roche	L01XC14	0	Oct 2014			Breast neoplasm	P	Money-back guarantee
Italy	Mozobil	Plerixafor	Genzyme	L03AX16	1	Dec 2011		July 2015	Lymphoma and multiple myeloma, in combination with G-CSF for	P	Money-back guarantee
Italy	Nexavar	Sorafenib	Bayer	L01XE05	1	July 2008	Feb 2016		Hepatocellular carcinoma	P	Money-back guarantee
Italy	Adcetris	Brentuximab	Takeda	L01XC12	1	July 2014			Hodgkin Disease, relapsed after ASCT or refractory to ASCT	F	Free/discounted treatment initiation
Italy	Adcetris	Brentuximab	Takeda	L01XC13	1	July 2014			Non-Hodgkin lymphoma	F	Free/discounted treatment initiation
Italy	Arzerra	Ofatumumab	Novartis	L01XC10	1	June 2011			Chronic lymphocytic B-cell leukemia	F	Free/discounted treatment initiation
Italy	Avastin	Bevacizumab	Roche	L01XC07	0	Oct 2005		July 2014	Colorectal carcinoma, first line	F	Free/discounted treatment initiation
Italy	Caprelsa	Vandetanib	AstraZeneca	L01XE12	0	July 2013			Thyroid neoplasms	F	Free/discounted treatment initiation

Italy	Dacogen	Decitabine	Jansen-cilag	L01BC08	1	Nov 2014			Acute myeloid leukemia in patients older than 65 years	F	Free/discounted treatment initiation
Italy	Erivedge	Vismodegib	Genentech	L01XX43	0	April 2015			Symptomatic, metastatic basal cell carcinoma and locally advanced basal cell carcinoma where surgery or radiation are inappropriate	F	Free/discounted treatment initiation
Italy	Nexavar	Sorafenib	Bayer	L01XE05	1	Nov 2006	Feb 2016		Renal cell carcinoma	F	Free/discounted treatment initiation
Italy	Removab	Catumaxomab	Neovi Biotech	L01XC09	0	Oct 2011		Feb 2016	Maligne Ascites positive for epCAM	F	Free/discounted treatment initiation
Italy	Revlimid	Lenalidomide	Celgene	L04AX04	1	March 2008			Myelodysplastic syndromes	F	Free/discounted treatment initiation
Italy	Sprycel	Dasatinib	BMS	L01XE06	1	May 2007		Oct 2008	Myelogenous, Chronic, BCR-ABL Positive Precursor Cell Lymphoblastic Leukemia-Lymphoma	F	Free/discounted treatment initiation
Italy	Sprycel	Dasatinib	BMS	L01XE06	1	Dec 2011			Myelogenous, Chronic, BCR-ABL Positive Precursor Cell Lymphoblastic Leukemia-Lymphoma, first line	F	Free/discounted treatment initiation
Italy	Tafinlar	Dabrafenib	Novartis	L01XE23	0	Nov 2014		April 2015	Melanoma positive for BRAF V-600	P	Money-back guarantee
Italy	Sutent	Sunitinib	Pfizer	L01XE04	0	Oct 2007			Advanced renal cell carcinoma	F	Free/discounted treatment initiation
Italy	Tarceva	Erlotinib	Roche	L01XE03	0	July 2006		May 2013	Non-small-cell lung carcinoma, first line	F	Free/discounted treatment initiation
Italy	Tarceva	Erlotinib	Roche	L01XE03	0	May 2013		July 2015	Non-small-cell lung carcinoma, further lines	F	Free/discounted treatment initiation

Italy	Tasigna	Nilotinib	Novartis	L01XE08	1	Dec 2011		Chronic myelogenous Leukemia positive for BCR-ABL , first line	F	Free/discounted treatment initiation
Italy	Torisel	Temsirolimus	Pfizer	L01XE09	0	Aug 2011		Mantle cell lymphoma, refractory or relapsed	F	Free/discounted treatment initiation
Italy	Velcade	Bortezomib	Janssen-cilag	L01XX02	0	July 2009	July 2015	Multiple myeloma	F	Free/discounted treatment initiation
Italy	Vidaza	Azacitidina	Celgene	L01BC07	1	Nov 2010		Myelodysplastic Syndromes	F	Free/discounted treatment initiation
Italy	Vidaza	Azacitidina	Celgene	L01BC07	1	Nov 2010		Chronic myelomonocytic leukaemia	F	Free/discounted treatment initiation
Italy	Vidaza	Azacitidina	Celgene	L01BC07	1	Nov 2010		Acute myeloid leukaemia	F	Free/discounted treatment initiation
Italy	Votrient	Pazopanib	GSK	L01XE11	0	July 2013		Soft tissue sarcoma in advanced stage, previously treated with chemotherapy	F	Free/discounted treatment initiation
Italy	Tasigna	Nilotinib	Novartis	L01XE08	1	Aug 2008		Chronic myelogenous leukemia, positive for BCR-ABL and resistant or intolerant to imatinib, second line	P	Money-back guarantee
Italy	Votrient	Pazopanib	GSK	L01XE11	0	May 2011		Renal cell carcinoma, first line and second line treatment for advanced stage previously treated with cytokine therapy	F	Free/discounted treatment initiation
Italy	Torisel	Temsirolimus	Pfizer	L01XE09	0	Oct 2008		Renal cell carcinoma	P	Money-back guarantee
Italy	Xtandi	Enzalutamide	Astellas Pharma	L02BB04	0	Dec 2014		Prostatic neoplasms, metastatic and resistant to castration and docetaxel	F	Free/discounted treatment initiation

Italy	Tyverb	Lapatinib	GSK	L01XE07	0	June 2011			Breast neoplasms, 1st line	P	Money-back guarantee
Italy	Tyverb	Lapatinib	GSK	L01XE07	0	June 2009			Breast neoplasm, 2nd line	P	Money-back guarantee
Italy	Zaltrap	Aflibercept	Regeneron	L01XX44	0	Oct 2014			Metastatic colon cancer, resistant or progressed after oxaliplatine containing regimen, in combination with irinotecan-based therapy/ 5-fluorouracil/folinic acid	F	Free/discounted treatment initiation
Italy	Zytiga	Abiraterone acetate	Jansen-cilag	L02BX03	0	April 2013			Metastatic castration-resistant prostate cancer in advanced stage or after docetaxel-based chemotherapy, in combination with prednisone or prednisolone, 1st line	F	Free/discounted treatment initiation
Italy	Vectibix	Panitumumab	Amgen	L01XC08	0	Jan 2009	June 2014		Monotherapy for metastatic colon cancer with expression of EGFR after fluoropyrimidine, oxaliplatine and irinotecan in tumours with WT KRAS	P	Money-back guarantee
Italy	Xalkori	Crizotinib	Pfizer	L01XE16	0	April 2015			Non-small-cell lung carcinoma positive for ALK in advanced stage	P	Money-back guarantee
Italy	Yervoy	Ipilimumab	Bristol Myers Squibb	L01XC11	0	March 2013		Sep 2014	Advanced (inoperable or metastatic) melanoma	P	Money-back guarantee
Italy	Yondelis	Trabectedin	Pharma Mar	L01XC01	1	Feb 2009			Advanced soft tissue sarcoma, unsuitable or irresponsive to antracycline and ifosfamide	P	Money-back guarantee
Italy	Yondelis	Trabectedin	Pharma Mar	L01XC01	1	April 2011			Platina-sensitive ovarian carcinoma in, relapsed, combination with pegylated liposomal doxorubicin	P	Money-back guarantee
Italy	Zelboraf	Vemurafenib	Roche	L01XE15	0	June 2013		Nov 2015	Monotherapy for irresistible or metastatic melanoma with BRAF V600 mutation	P	Money-back guarantee

Italy	Zytiga	abiraterone acetate	Jansen-cilag	L02BX03	0	April 2013	Metastatic castration-resistant prostate cancer in after adrogendeprivate therapy, 2nd line	P	Money-back guarantee
Scotland	Arzerra	Ofatumumab	Novartis Pharmaceuticals UK Ltd	L01XC10	1	April 2015	Chronic lymphocytic leukemia, not previously treated or not eligible for fludarabine based therapy, in combination with chlorambucil or bendamustine	F	Discount
Scotland	Velcade	bortezomib	Janssen-Cilag	L01XX02	0	Nov 2009	Monotherapy for the treatment of progressive multiple myeloma, treated with at least one prior theapy and already undergone or unsuitable for bone marrow transplantation	P	Outcomes guarantee
Scotland	Avastin	Bevacizumab	Roche	L01XC07	0	Aug 2015	Platinum resistant recurrent epithelial ovarian, fallopian tube, or primay peritoneal cancer, who received no more than two prior chemotherapy regimes and who have not received prior therapy with bevacizumab or other vascular endothelial growth factor inhibitors or VEGF receptor targeted agents, in combination with paclitaxel, topotecan, or pegylated liposomal doxorubicin	F	Discount
Scotland	Bosulif	Bosutinib	Pfizer	L01XE14	0	Feb 2015	Chronic phase, accelerated phase and blast phase philadelphia positive chronic myelogenous leukemia, previously treated with one or more tyrosine kinase inhibitors and for whom imatinib, nilotinib and dasatinib are not conisdered appropriate treatment options	F	Discount
Scotland	Erbutix	Cetuximab	Merck Serono	L01XC06	0	Jan 2010	Epidermal growth factor receptor expressing, Kirsten rat sarcoma wild-type metastatic colorectal cancer in combination with chemotherapy	F	Discount
Scotland	Erbutix	Cetuximab	Merck Serono	L01XC06	0	Jan 2015	EGFR expressing, RAS wildtype metastatic colorectal cancer	F	Discount

Scotland	Firmagon	Degarelix	Ferring Pharmaceuticals	L02BX02	0	Nov 2010	Advanced hormone-dependent prostate cancer	F	Discount
Scotland	Giotrif	Afatinib	Boehringer-Ingelheim	L01XE13	0	March 2014	EGFR tyrosine kinase inhibitor naive adults, with locally advanced or metastatic NSCLC with activating EGFR mutations	F	Discount
Scotland	Halavan	Eribulin	Eisai	L01XX45	0	Feb 2016	Locally advanced or metastatic breast cancer , progressed after at least one chemotherapeutic regimen for advanced disease and prior treated with anthracycline and a taxane in either the adjuvant or metastatic setting unless not suitable	F	Discount
Scotland	Imnovid	Pomalidomide	Celgene	L04AX06	1	Dec 2014	Relapsed and refractory multiple myeloma after at least two prior treatment regimes, including lenalidomide and bortezomib, and demonstraed disease progression on the lastest therapy, in combination with dexamethasone	F	Discount
Scotland	Inlyta	Axitinib	Pfizer	L01XE17	0	Nov 2013	Advanced renal cell carcinoma after failure of prior treatment with sunitinib or a cytokine	F	Discount
Scotland	Iressa	Gefitinib	AstraZeneca	L01XE02	0	Nov 2015	Locally advanced or metastatic non-small cell lung cancer (NSCLC) with activating mutations of epidermal growth factor receptor tyrosine kinase (EGFR-TK)	F	Discount
Scotland	Votrient	Pazopanib	GSK	L01XE11	0	Feb 2013	First-line treatment of advanced renal cell carcinoma for patients who have received prior cytokne therapy for advanced disease	F+P	Discount + coverage with evidence development
Scotland	Keytruda	Pembrolizumab	Merck Shapr and Dhome	L01XC18	0	Oct 2015	Monotherapy for the treatment of advanced (unresectable or metastatic) melanoma, previously untreated with ipilimumab	F	Discount
Scotland	Mabthera	Rituximab	Roche	L01XC02	0	July 2014	Non-hodgkin's lymfoma	F	Discount

Scotland	Mepact	Mifamurtide	takeda	L03AX01	1	Aug 2011	high-grade resectable non-metastatic osteosarcoma after macroscopically complete surgical resection, in combination with post-operative multi-agent chemotherapy	F	Discount
Scotland	Nexavar	Sorafenib	Bayer	L01XE05	1	June 2015	Progressive, locally advanced or metastatic, differentiated thyroid carcinoma, refractory to radioactive iodine	F	Discount
Scotland	Stivarga	Regorafenib	Bayer	L01XE21	0	April 2015	Unresectable or metastatic gastrointestinal stromal tumors who progressed on or are intolerant to prior treatment with imatinib and sunitinib	F	Discount
Scotland	Sutent	Sunitinib	Pfizer	L01XE04	0	Oct 2009	Unresectable and metastatic malignant GIST after failure of imatinib mesilate treatment due to resistance or intolerance	F	Free/discounted treatment initiation
Scotland	Sutent	Sunitinib	Pfizer	L01XE04	0	April 2011	Treatment of unreseactable or metastatic, well-differentiated pancreatic neuroendocrine tumours with disease progression in adults.	F	Free/discounted treatment initiation
Scotland	Tafinlar	Dabrafenib	GSK	L01XE23	0	March2015	Unresectable or metastatic melanoma with a BRAF V600 mutation	F	Discount
Scotland	Tarceva	Erlotinib	Roche	L01XE03	0	Jan 2012	Locally advanced or metastatic non-small cell lung cancer with epidermal growth factor receptor activating mutations, first line	F	Discount
Scotland	Tasigna	nilotinib	Novartis	L01XE08	1	Jan 2012	Newly diagnosed philadelphia chromosome positive chronic myelogenous leukeamia in the chronic phase	F	Discount
Scotland	Vargatef	nintedanib	Boehringer Ingelheim	L01XE31	0	April 2015	Locally advanced, metastatic or locally recurrent NSCLC of adenocarcinoma tumor histology after firstline therapy, in combination with docetaxel	F	Discount

Scotland	Vidaza	Azacitidine	Celgene	L01BC07	1	Sep 2011	Chronic myelomonocytic leukemia or acute myeloid leukemia not eligible for haematopoetic stem cell transplantation	F	Discount
Scotland	Xalkori	Crizotinib	Pfizer	L01XE16	0	Oct 2013	Anaplastic lymphoma kinase (ALK)-positive advanced NSCLC, previously treated	F	Discount
Scotland	Xtandi	Enzolutamide	Astellas Pharma Ltd	L02BB04	0	Nov 2013	Metastatic castration-resistant prostate cancer (mCRPC) whose disease has progressed on or after docetaxel therapy	F	Discount
Scotland	Yervoy	Ipilimumab	Bristol Myers Squibb	L01XC11	0	April 2013	Treatment of advanced (unresectable or metastatic) melanoma after prior therapy	F	Discount
Scotland	Zaltrap	Aflibercept	Sanofi	L01XX44	0	Feb 2014	Metastatic colorectal cancer (mCRCC) that is resistant to or has progressed after an oxaliplatin-containing regimen, in combination with irinotecan/5-fluorouracil/folinic acid chemotherapy	F	Discount
Scotland	Zelboraf	Vemurafenib	Roche	L01XE15	0	Dec 2013	Unresectable or metastic melanoma, positive for BRAF V600 mutation, first line	F	Discount
Scotland	Zydelig	Idelalisib	Gilead	L01XE15	0	Feb 2015	Chronic lymfocytic leukemia, in combination with rituximab	F	Discount
Scotland	Zydelig	idelalisib	Gilead	L01XE15	0	March 2015	Follicular lymfoma that is refractory to two prior lines of treatment	F	Discount
Scotland	Zykadia	Ceritinib	Novartis europpharm Limited	L01XE28	0	Nov 2015	Advanced non small cell lung cancer (NSCLC), positive for anaplastic lymphoma kinase (ALK), previously treated with crizotinib	F	Discount
Scotland	Zytiga	Abiraterone Acetate	Janssen-cilag	L02BX03	0	Sept 2015	Metastatic prostate cancer resistant to castration, progressed on or after docetaxel based treatment regimen, in combination with prednison or prednisolon	F	Discount

Sweden	Mekinist	Trametinib	Novartis	L01XE25	0	July 2016	dec/17	Melanoma, combination therapy	F	Money-back guarantee
Sweden	Xtandi	Enzalutamide	Astellas Pharma	L02BB04	0	July 2015	July 2017	Prostate cancer	F	Patient utilisation cap
Sweden	Zykadia	Ceritinib	Novartis	L01XE28	0	Dec 2015	June 2017	Non-Small cell lung cancer	F	Patient utilisation cap
Sweden	Zytiga	Abiraterone acetate	Janssen-Cilag	L02BX03	0	June 2015	June 2017	Prostate cancer	F	Patient utilisation cap
Wales	Stivarga	Regorafenib	Bayer	L01XE21	0	July 2015		Treatment of adult patients with unresectable or metastatic gastrointestinal stromal tumours (GIST) who progressed on or are intolerant to prior treatment with imatinib and sunitinib	Confidential	Confidential
Wales	Velcade	Bortezomib	Janssen-Cilag	L01XX02	0	October 2014		n combination with pegylated liposomal doxorubicin or dexamethasone for the treatment of adult patients with progressive multiple myeloma who have received at least 1 prior therapy and who have already undergone or are unsuitable for haematopoietic stem cell transplantation	P	Money-back guarantee
Wales	Degarelix	Firmagon	Ferring Pharmaceuticals	L02BX02	0	Dec 2012		Advanced hormone-dependent prostate cancer	F	Discount
Wales	Imnovid	Pomalidomide	Celgene	L04AX06	1	Aug 2015		Relapsed and refractory multiple myeloma who have received at least two prior treatment regimens, including both lenalidomide and bortezomib, and have demonstrated disease progression on the last therapy, in combination with dexamethasone	F	Discount

Wales	Mabthera	Rituximab	Roche	L01XC02	0	Jan 2015	Non-Hodgkin's lymphoma, treatment of previously untreated patients with stage III-IV follicular lymphoma in combination with chemotherapy; maintenance therapy for the treatment of follicular lymphoma patients responding to induction therapy; and treatment of patients with CD20 positive diffuse large B cell non-Hodgkin's lymphoma in combination with CHOP (cyclophosphamide, doxorubicin, vincristine, prednisolone) chemotherapy	F	Discount
Wales	Tyvverb	Lapatinib	GSK	L01XE07	0	Aug 2013	Breast cancer, whose tumours overexpress HER2 (ErbB2), in combination with capecitabine for patients with advanced or metastatic disease with progression following prior therapy, which must have included anthracyclines and taxanes and therapy with trastuzumab in the metastatic setting	F	Discount
Wales	Votrient	Pazopanib	GSK	L01XE11	0	Dec 2013	Advanced soft tissue sarcoma who have received prior chemotherapy for metastatic disease or who have progressed within 12 months after (neo) adjuvant therapy	F	Discount + rebate
