# Supplementary material for: Managed Entry Agreements for Oncology Drugs: Lessons from the European Experience to Inform the Future
Source: Front Pharmacol. 2017 Apr 4;8:171. doi: 10.3389/fphar.2017.00171 (PMC5378787; doi:10.3389/fphar.2017.00171)
Supplement: Supplementary file 2 [file DataSheet2.pdf]

Belgium

The Commission of Reimbursement of Medicines (CRM) is composed by academics, representatives of insurance companies, representative of pharmacist associations, representatives of physician associations, representatives of pharmaceutical industry associations and a representative for the Minister of Social Affairs, the Minister of Health affairs, the Minister of Economic affairs and a representative of the National Institute of Health and Disability Insurance (NIHDI). The CRM evaluates the reimbursement application and formulates an advice to the Minister. MEA are allowed according to Article 81, introduced in the Royal Decree in 2010. Between 2010 and 2014, the company could request negotiation of a MEA when the advice of the CRM was negative or inconclusive. In 2014, Article 81 bis was introduced in the law and from then on, it was no longer possible to request negotiation of a MEA in case of a negative advice. According to article 81 bis, a MEA can be requested by the CRM, or can be requested by the company in case that the advice of the CRM was inconclusive. The content of the MEA is negotiated in a working party in which the Minister of Social affairs, the Minister of Economic Affairs, the Minister of Budgetary Affairs, the insurance companies, the pharmaceutical industry association and the MAH is represented (13).

The Netherlands

The National Health Care Institute (Zorginstituut Nederland) is involved in maintaining the quality, accessibility and affordability of healthcare. The National health care institute provides an independent advice to the Minister of public Health, Welfare and Sport about the insurance of new medical interventions for inpatient and outpatient care based on therapeutic value, budget impact and cost-effectiveness. Specialty medicines for intramural care are automatically available after marketing authorization. Evaluation for specialty medicines by the Institute is limited to medicines that pose budgetary risks to the hospital, based on notification by the manufacturer and monitoring by the Institute. The Institute can advise to negotiate a financial arrangement for intramural care since 2012. After the Institute has evaluated the drug, the negotiation is hold between the Office of Financial Agreements, which is part of the Ministry of Health, and the company. Before the negotiation, the professional associations of physicians can be consulted by the Office. In 2015, the application of financial agreements was extended to inpatient drugs as well.

Czech Republic

Since 2008, the State Institute for Drug Control (SUKL) is responsible for reimbursement of out-patient (not hospital only) drugs and is responsible for price setting of both out-patient and hospital only products. The ex-factory price is set based on the average of 3 lowest ex-factory prices in the reference countries (all EU countries except Austria, Bulgaria, Cyprus, Czech Republic, Estonia, Germany,

Luxembourg, Malta and Romania). For highly innovative drugs, the average price found in at least 2 reference states can be used. A system of internal reference price setting is applied for reimbursement of out-patient drugs. Reference groups are composed based on similar effectiveness, safety profile, clinical use and therapeutically interchangeability. The lowest EU price of a pharmaceutical within a reference group determines the reimbursement price. Premium reimbursement can be granted to drugs that have better effectiveness, safety profile or compliance rates. For in-patient drugs, the Insurance Funds Union evaluates the efficacy, cost-effectiveness and budget impact. Negotiation of MEA is not specifically included in the legislation, although applied by the Insurance Funds Union in case of unfavorable budget impact or cost-effectiveness.

Sweden

Since 2002, TLV acts as an independent agency responsible for reimbursement and price setting of out-patient pharmaceuticals, while twenty one county councils negotiates/tender prices for in-patient pharmaceuticals. County councils are associated by Swedish Association of Local Authorities and Regions (SALAR, SKL). The Supreme Administrative Court has clarified that TLV does not have the legal right to prohibit pharmaceutical companies and County Councils from negotiating discounts on out-patient pharmaceuticals included in the national reimbursement scheme. MEA can be concluded between the county councils and the MAH through a new form of collaboration which is facilitated by TLV and a working group in which the county councils are coordinated by SALAR. Since 2015, the TLV is also involved in evaluation of in-patient medicines, in order to structure the recommendation towards county councils and facilitate homogenous access to care across county councils. Pharmaceuticals eligible for this process will be selected through horizon scanning and presented to the TLV by the New Therapy (NT) council. The NT council will transfer the application to the TLV, and will formulate a recommendation towards the county councils based on the TLV evaluation

England & Wales

MEA are applied under the term patient access schemes (PAS) in England and Wales. PAS were introduced in the Pharmaceutical Price Regulation Scheme (PPRS) in 2009 in order to allow better reflection of the value of medicines and to improve the cost-effectiveness of innovative drugs within the context of an appraisal by the National Institute of Health and Care Excellence (NICE) (10;11). The current framework for PAS is set out in the 2014 PPRS. A MAH can propose a PAS to the Department of Health when a medicine is due to be appraised by NICE. The PPRS says that PAS should be proposed either at the outset or the end of the NICE appraisal process, though simple discount proposal may exceptionally be accepted at other stages of the process. The NICE PAS Liaison Unit (PASLU) is in charge

of evaluating whether a PAS is feasible within England and Wales and providing advice to the Department of Health. Where a PAS proposal agreed by the Department of Health goes on to form part of positive NICE guidance recommending the relevant medicines within the PAS, the PAS applies automatically within England and Wales. Where the NHS in wales is separately evaluating a product that is not due to be appraised by NICE, Wales PAS (WPAS) can be proposed by a MAH and agreed with the Welsh Government, after evaluation by the Patient Access Schemes Wales Group (PASWG). The PASWG was set up in 2011 and had the task to consider the feasibility, workability and acceptability of PAS within NHS Wales and to provide advice to the Welsh Government. AWMSG advice, with or without a WPAS, will however be replaced by that of a NICE recommendation in case they subsequently issue final advice for the same technology.

Scotland

The Scottish Medicines Consortium (SMC) evaluates of the clinical and cost effectiveness of all newly licensed drugs and advises Health Boards and their Area Drug and Therapeutics Committees, on their use in NHS Scotland. MEA are applied by the Scottish Medicines Consortium (SMC) under the term Patient Access Scheme (PAS) since 2011. PAS are defined as a scheme proposed by a pharmaceutical company in order to improve the cost-effectiveness of a medicine and enable patients to receive access to cost-effective innovative medicines. The Patient Access Schemes Assessment Group (PASAG) advices the NHS Scotland about the acceptability and feasibility of PAS submissions. PASAG operates separately from SMC to maintain the integrity and independence of the assessment process. Standard process is for companies to propose a PAS before the medicine is assessed by SMC; however in the case of end of life medicines and orphan medicines, PAS can also be proposed by companies after the New Drug Committee (NDC) defines its provisional advice. The New Drugs Committee (NDC) examines the submission with the assistance of medical, pharmaceutical, statistics and economics experts and provides provisional advice to the SMC and the pharmaceutical company.

Italy

The Italian Medicines Agency (AIFA) started its first contract in July 2006 (11). MEA can be requested by the pharmaceutical company or by the Technical Scientific Committee (CTS), who delivers an advice on reimbursement based on the marketing authorization application. The negotiation on MEA is held within the setting of price negotiation between the company and Prices and Reimbursement Committee (CPR). No other parties are involved in the negotiation, although one can seek for external advice from scientific committees.

France

After MA, the medical evidence of drugs is assessed by the Haute Autorité de Santé (HAS) before inclusion on a positive list for reimbursement of hospital pharmacy products or community pharmacy products. The Economic Committee of Health Products (CEPS) negotiates the price of new drugs with the pharmaceutical company, taking into account the evaluation by HAS. For products with high medical benefit, price consistency with other European countries is guaranteed, meaning that the price will be no lower than the lowest price in the four main comparable markets. A safeguard clause secures monetary contribution from pharmaceutical companies if the growth rate of turnover of drugs for community pharmacy, outpatient care in the hospital and hospital care exceed a certain rate.
